# Tailored therapy guided by multichannel intraluminal impedance pH monitoring for refractory non-erosive reflux disease

**DOI:** 10.1038/cddis.2017.436

**Published:** 2017-09-07

**Authors:** Nunzio Ranaldo, Giuseppe Losurdo, Andrea Iannone, Mariabeatrice Principi, Michele Barone, Massimo De Carne, Enzo Ierardi, Alfredo Di Leo

**Affiliations:** 1 Gastroenterology Section, Department of Emergency and Organ Transplantation, Piazza Giulio Cesare, University of Bari, Bari, Italy; 2 Gastroenterology Section, IRCCS 'De Bellis', Castellana Grotte (BA), Italy

## Abstract

A relevant percentage of non-erosive reflux disease (NERD) is refractory to proton pump inhibitors (PPIs) treatment. Multichannel intraluminal impedance pH (MII-pH) monitoring should give useful pathophysiological information about refractoriness. Therefore, our aim was to assess whether this technique could be useful to guide a 'tailored' therapy in refractory NERD. We retrospectively recruited NERD patients undergoing MII-pH monitoring for unsuccessful treatment. All patients had undergone upper endoscopy, and those with erosive esophagitis were excluded. No patient received PPI during MII-pH monitoring. Subjects were subgrouped into three categories: acid reflux, non-acid reflux and functional heartburn. MII-pH-guided therapy was performed for 4 weeks as follows: patients with acid reflux received PPI at double dose, patients with non-acid reflux PPI at full dose plus alginate four times a day and patients with functional heartburn levosulpiride 75 mg per day. A visual analog scale (VAS) ranging from 0 to 100 mm was administered before and after such tailored therapy to evaluate overall symptoms. Responders were defined by VAS improvement of at least 40%. Sixty-nine patients with refractory NERD were selected (female–male ratio 43 : 26, mean age 47.6±15.2 years). Overall effectiveness of tailored therapy was 84% without statistical difference among subgroups (88.5% acid reflux, 92% non-acid reflux, 66.6% functional heartburn; *P*=0.06). Univariate analysis showed that therapy failure directly correlated with functional heartburn diagnosis (OR=4.60) and suggested a trend toward a negative correlation with smoking and a positive one with nausea. However, at multivariate analysis, these parameters were not significant. Functional heartburn experienced a lower median percent VAS reduction than acid reflux (52.5% *versus* 66.6%, *P*<0.01) even if equal to non-acid reflux (66.6%). In conclusion, a tailored approach to refractory NERD, guided by MII-pH monitoring, demonstrated to be effective and should be promising to cure symptom persistence after conventional therapy failure. Nevertheless, standardized guidelines are advisable.

Gastroesophageal reflux disease (GERD) is a chronic disorder characterized by abnormal exposure of esophageal mucosa to gastric content, resulting in different troublesome symptoms, with heartburn and regurgitation being the most common.^1^ Nevertheless, the clinical picture of GERD encompasses both typical (heartburn, epigastric pain, regurgitation, belching, nausea, vomit, sensation of 'heavy stomach' or 'feeling full quickly') and atypical symptoms (cough, hoarse voice, globus, sore throat or ear pain).

About the 40% of the US population is affected by GERD-related symptoms once a month and 20% once a week.^2, 3^ Patients with GERD are commonly classified into two categories: non-erosive reflux disease (NERD) or erosive esophagitis. Community-based European studies found a NERD prevalence of up to 70%.^4, 5^

The treatment of NERD consists mainly in modifications of dietary and lifestyle habits and proton pump inhibitor (PPI) assumption. Despite these cautions, there is no improvement of the symptoms in a relevant percentage of patients (refractory NERD). In particular, refractoriness is defined by the persistence of typical symptoms despite receiving a full dose of PPI for at least 12 weeks.^6^ However, some authors believe that lack of clinical response after a 8-week course of PPI may be sufficient to define refractoriness for NERD.^7, 8^ Refractory NERD is often a therapeutic challenge. Indeed, in a systematic review, pooled rate of symptomatic response to PPI was 36.7% (95% confidence interval: 34.1–39.3) in NERD and 55.5% (95% confidence interval: 51.5–59.5) in erosive esophagitis.^9^ Several mechanisms may be invoked to explain refractoriness: (i) presence of non-acid/weakly acid reflux, (ii) low tone of lower esophageal sphincter, (iii) poor esophageal clearance, (iv) existence of an acid pocket located close to the gastroesophageal junction, (v) functional heartburn and (vi) poor compliance to the therapy or inadequate optimization of the treatment.^6^

Multichannel intraluminal impedance pH (MII-pH) monitoring has been proposed as a tool to improve the management of NERD. Indeed, compared with traditional pH monitoring, it allows the identification of both acid and non-acid reflux episodes, thus leading to differentiate NERD subtypes (i.e. acid and non-acid) and functional heartburn.^10, 11^ In the setting of refractory NERD, MII-pH monitoring could be a useful device to understand the reasons of therapy failure, thus addressing a 'tailored' treatment.^12, 13^ However, few reports have systematically applied MII-pH monitoring to optimize refractory NERD treatment.^13^

In the present study, we retrospectively investigated whether a MII-pH-guided 'tailored strategy' could have been beneficial in patients with refractory NERD.

## Results

### Success rate

**Figure 1:**
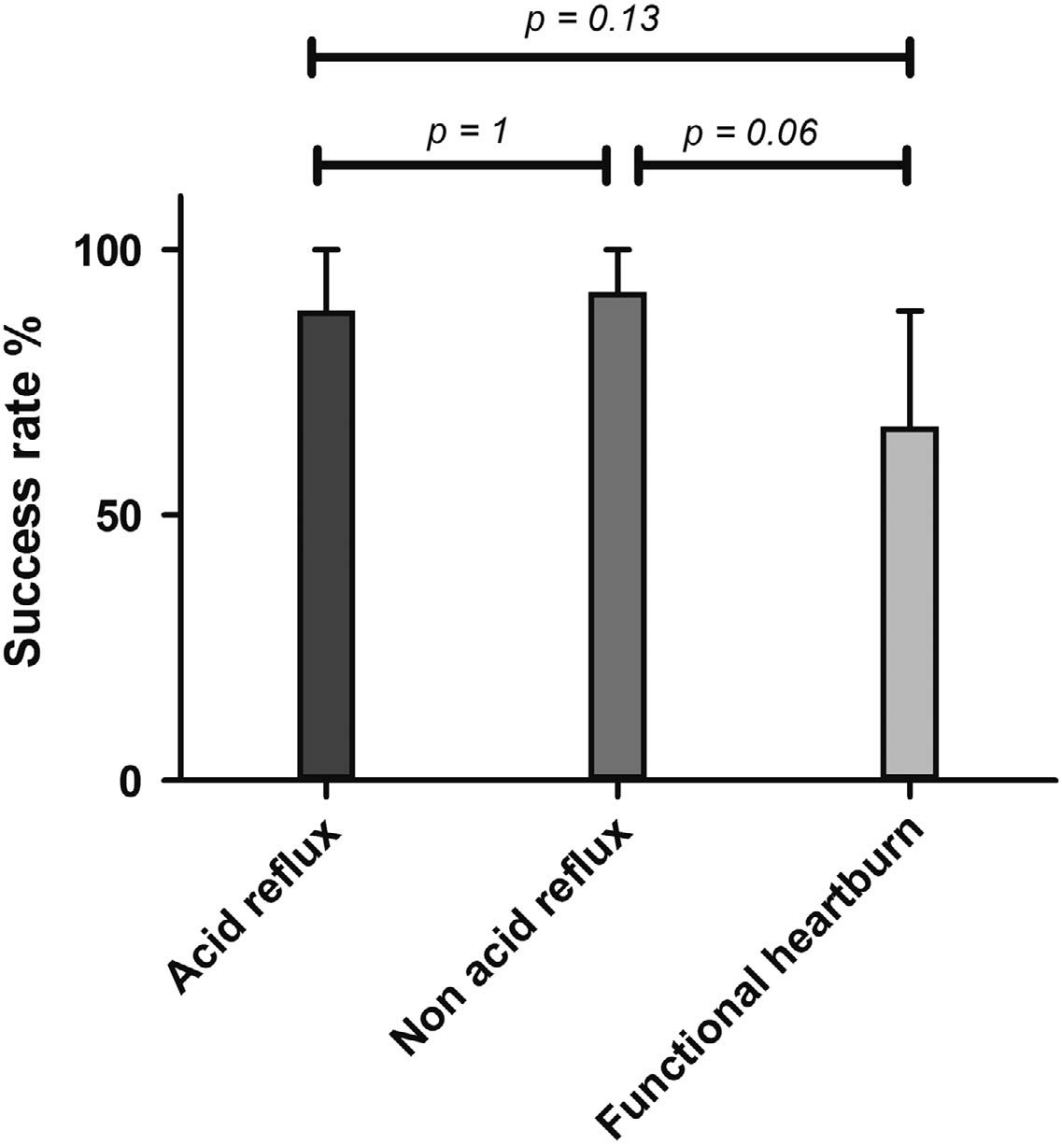
Success rates of tailored therapy according to the NERD subtypes after MII-pH monitoring. A *χ*^2^ test for trend was used. The bars represent the percentage of success, and the error bars the 95% confidence interval

Seventy-two patients were eligible according to inclusion criteria; 69 of them accepted to participate to the study. Enrolled patients showed a female–male ratio of 43 : 26 and a mean age of 47.6±15.2 years). Twenty-six had acid reflux, 25 non-acid reflux and 18 functional heartburn. Tailored therapy was effective in 58 out of 69 patients, with a success rate of 84.0% (95% CI: 75.4–92.6). The effectiveness of tailored therapy for acid reflux, non-acid reflux and functional heartburn was, respectively, 88.5% (95% CI: 76.2–100), 92.0% (95% CI: 81.4–100) and 66.6% (95% CI: 44.8–88.4), with a nonsignificant trend (*P*=0.06). This borderline trend for functional heartburn was confirmed in direct comparisons against acid reflux (*P*=0.13) and non-acid reflux (*P*=0.06), as graphically shown in Figure 1.

### Variations in VAS score

**Figure 2:**
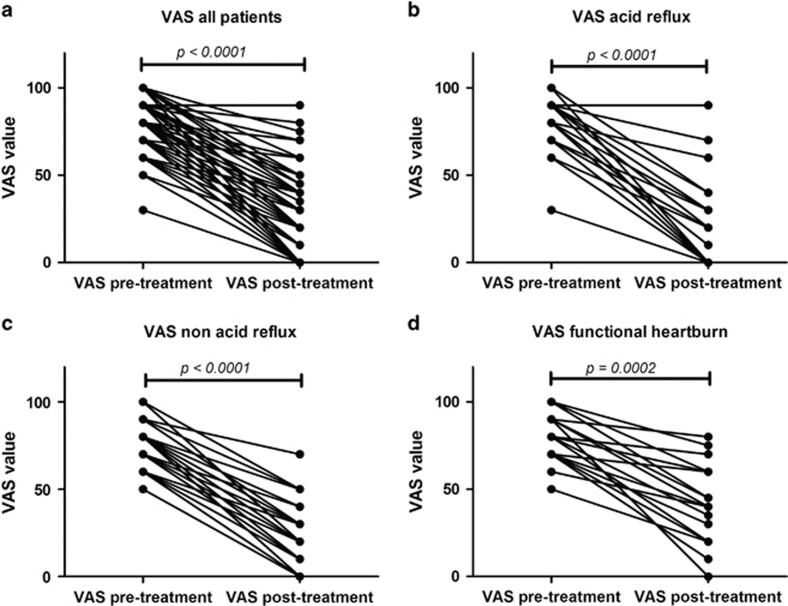
Variations in VAS score before and after the tailored treatment, for all patients (**a**) and patients with acid reflux (**b**), non-acid reflux (**c**) and functional heartburn (**d**)

In our overall cohort, median baseline visual analog scale (VAS) score was 80 mm and interquartile range was 20 mm, whereas, after the treatment, the median was 30 mm and interquartile range was 30 mm, with a significant reduction (*P*<0.0001). In patients with acid reflux, VAS score dropped from median 80 mm and interquartile range 20 mm to median 20 mm and interquartile range 30 mm after the therapy (*P*<0.0001). Similarly, patients with non-acid reflux experienced a decrease in VAS value from median 80 mm and interquartile range 25 mm to median 20 mm and interquartile range 30 mm after the treatment (*P*<0.0001). Subjects with functional heartburn reported a statistically significant drop (*P*=0.0002) in VAS scale from median 80 mm and interquartile range 20 mm to median 40 mm and interquartile range 40 mm. These variations in VAS scale before and after the treatment are graphically represented in Figure 2.

**Figure 3:**
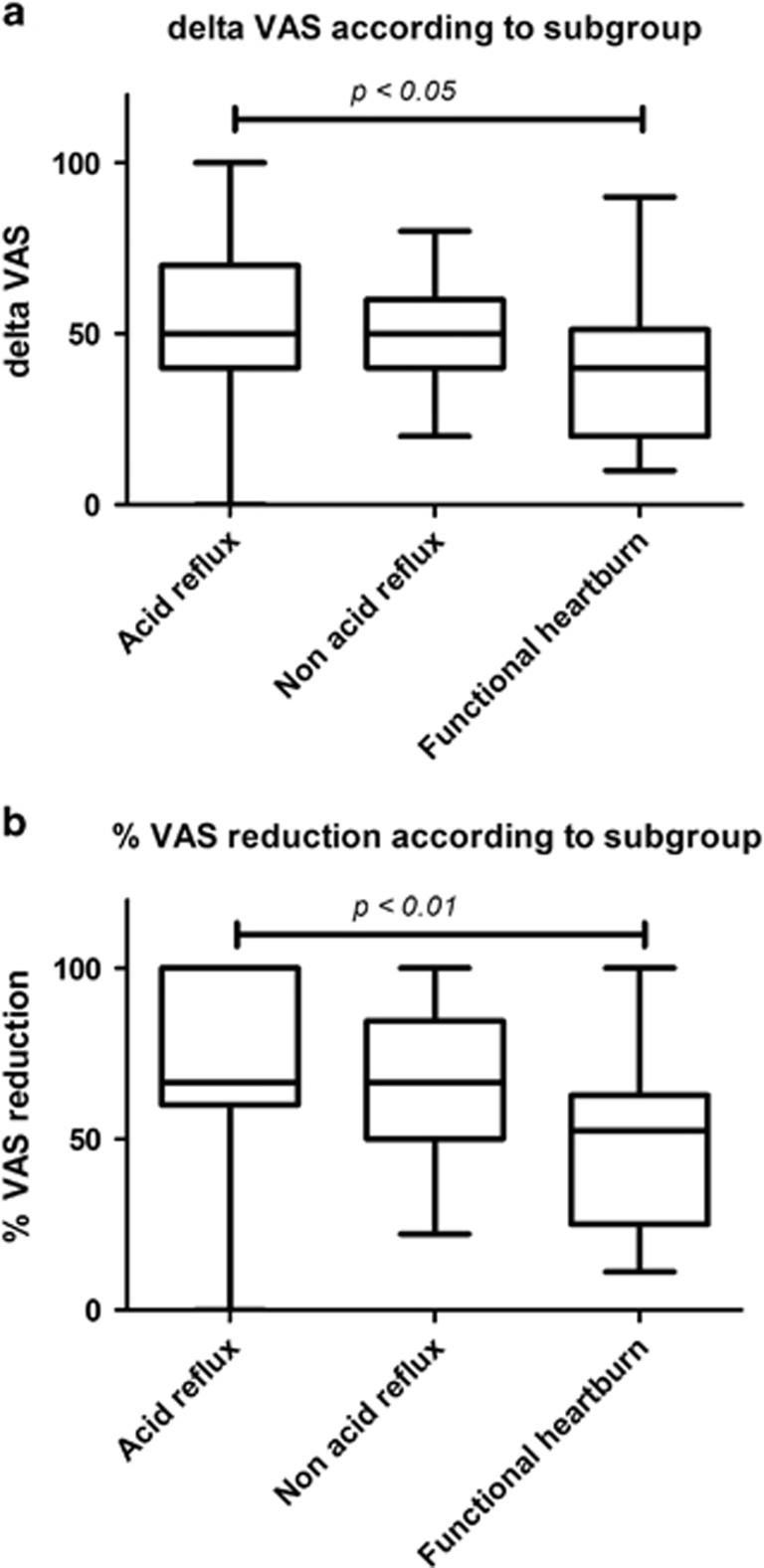
In (**a**), mean delta VAS for each subgroup patients (acid, non-acid reflux and functional heartburn) are illustrated and compared by Kruskal–Wallis test with Dunn’s *post hoc* analysis. In (**b**), percent variations VAS for each subgroup patients are reported and compared by Kruskal–Wallis test with Dunn’s *post hoc* analysis. *P<0.05; **P<0.01. The figure reports boxplots, summarizing median, interquartile range, minimum and maximum

Therefore, we found a median VAS reduction (delta) of 50 mm and interquartile range of 20 mm. The delta VAS was lower in functional heartburn (median 40 mm, interquartile range 31.2 mm) compared with acid reflux (median 50 mm, interquartile range 30 mm, *P*<0.05), even if not different from non-acid reflux (median 50 mm, interquartile range 20 mm), as reported in Figure 3a.

We found an overall median percent reduction of VAS score of 62.5% and an interquartile range of 35.7%. The percent reduction of VAS score of functional heartburn (median 52.5%, interquartile range 37.9%) was lower than that of acid reflux (median 66.6%, interquartile range 40%, *P*<0.01), but similar to non-acid reflux (median 66.6%, interquartile range 34.5%), as shown in Figure 3b.

### Factors associated with tailored therapy failure

When responders were compared to non-responders (univariate analysis, reported in Table 1), we found that more patients were smokers among the responders in a nonsignificant trend (29.3% *versus* 0%, *P*=0.054). Smoking habits suggested a trend toward a negative correlation with therapy failure at univariate analysis. (OR=0.22; 95% CI: 0.07–1.00; *P*=0.054).

Nausea was more common among non-responders (54.5% *versus* 24.1%, *P*=0.06). Similar to smoking, at univariate analysis, we found a trend to correlate directly with therapy failure (OR=3.77; 95% CI: 0.99–14.27; *P*=0.06).

The diagnosis of functional heartburn was more common in non-responders than in responders (54.5% *versus* 20.7%, *P*=0.03). Patients with functional heartburn diagnosis showed a significant correlation with the risk of therapy failure (OR=4.60; 95% CI: 1.20–17.68; *P*=0.03), whereas the other MII-pH monitoring categories (acid and non-acid reflux) were not associated with unsuccessful treatment.

The type of PPI was neither associated with clinical response nor to therapy (*P*=0.15; Table 1).

At multivariate analysis (Table 2), smoking was not a statistically significant factor for therapy failure, with an OR=0.10 (95% CI: 0.002–18.9, *P*=0.99). Nausea and diagnosis of functional heartburn were not associated with failure, although a trend was evident both for nausea (OR=3.67; 95% CI: 0.85–15.88; *P*=0.08) and diagnosis of functional heartburn (OR=3.67; 95% CI: 0.85–15.88; *P*=0.08).

## Discussion

NERD is a frequent finding in clinical practice; however, refractoriness to PPI treatment is quite common. There is a clear evidence that the response rate to PPI administration is lower in subjects with NERD than in patients with erosive esophagitis.^9, 14^ Therefore, a systematic approach to refractory NERD is a therapeutic challenge of great interest. Up to now, MII-pH monitoring has been mainly applied to elucidate some pathophysiological aspects^6, 7, 15^ even if the investigation-related information may be useful for a tailored therapeutic approach.

Xiao *et al.*^13^ performed MII-pH monitoring on 39 subjects with refractory NERD and the test allowed to classify such patients into three main groups (acid and non-acid reflux functional heartburn). They reported an overall success rate of tailored therapy of 64.1%, with an effectiveness of 83.3% for acid reflux, 50% for non-acid reflux and 66.6% for functional heartburn, thus suggesting the usefulness of the test to guide the treatment.

In the present study, we used the same above-mentioned MII-pH-based classification of NERD^13^ even if our therapeutic approach was different with regard to functional heartburn (levosulpiride instead of paroxetine) and non-acid reflux (alginate instead of PPI double dose).

In functional heartburn, our choice was because of some concerns. We did not take into account the administration of an antidepressant drug to a group of patients with a benign and non-disabling disorder for possible side effects. Furthermore, similar to many other antidepressant agents, the effect of paroxetine is gradual and generally it takes some weeks to reveal its effectiveness.^16^ On the other hand, levosulpiride exerts effects on both psychological level and regulation of gastric and esophageal motility. It has been already tested on functional heartburn and has demonstrated an efficacy superior to metoclopramide and domperidone for this symptom relief as well as for postprandial bloating and epigastric pain improvement.^17, 18^ Additionally, Lozano *et al.*^19^ showed that a 15-day course of levosulpiride reduced the symptom score for functional heartburn by 50%, and the prolongation of therapy to 30 days led to symptom disappearance. Similarly, Mearin *et al.*^20^ demonstrated that this drug reduced the symptom score of 79.9% in a group of patients with dismotility-like functional heartburn. In the present experience, we found that this strategy was effective in two-thirds of patients, a value that is comparable with similar investigations. Despite the success rate was not inferior to the other NERD categories, we showed that the median improvement of VAS score was statistically lower than that of acid reflux group. This finding could be related to two main aspects: gastrointestinal functional disorders are frequently hard to treat and therapeutic response is subjective being affected by psychosomatic issues.^21, 22, 23, 24^ This fact was confirmed by the univariate analysis, where diagnosis of functional heartburn was correlated to a high risk of therapy failure. Nevertheless, we believe that a 66.6% rate of response in patients with previous diagnosis of refractoriness is still a relevant gain.

In refractory NERD patients with acid overexposure, PPI dose escalation seems to be the most effective strategy.^13^ A study investigating MII-pH monitoring for tailored therapy showed that 90.9% of patients with acid reflux improved after PPI dose doubling.^25^ Our experience confirmed such results, demonstrating a success rate of 88.5% using PPI double dose.

The pathophysiological mechanism of non-acid-reflux is an exaggerated relaxation of lower esophageal sphincter, with increased exposure to gastric content.^26^ For this reason, alginates, which create a mechanical barrier hampering the reflux of gastric content, may be useful for this purpose. In an Italian open-label study on refractory NERD, alginates were effective in controlling heartburn and reducing VAS score, despite that they did not decrease the number of non-acid reflux episodes.^27^ Similarly, several other studies demonstrated a good profile of alginates in reducing the burden of NERD symptoms.^28, 29^ Indeed, alginates have been demonstrated to neutralize also non-acid secretions such as pepsin and bile salts.^30^ Therefore, a simple inhibition of acid secretion by PPI, as proposed by Xiao *et al.*^13^ could not be appropriate in patients with non-acid reflux and this may be an explanation for their low success rate (50%). Conversely, herein we demonstrated that alginates plus a full dose of PPI are able to achieve a high response in non-acid reflux (92.0%).

At univariate analysis, a functional heartburn diagnosis was associated with tailored therapy failure, and this could be an expected finding, as we have already remarked.^21, 22, 23, 24^ Moreover, nausea showed a trend to directly correlate with therapy failure even if not statistically significant. The controversial relationship between smoking habit and refractory NERD stimulates some considerations. Several previous evidences have already stated that smoking is a risk factor for NERD^31, 32, 33^ and that its cessation leads to clinical improvement.^34^ However, in our cohort, we found that smokers seemed to have a higher chance of response to tailored therapy, and this observation is supported by some previous studies that have highlighted that non-smokers are at high risk of non-response to PPI therapy.^35, 36^ Finally, our multivariate analysis failed to find factors associated with tailored therapy response/failure.

Some limitations of the present study should be emphasized. The retrospective analysis of our results may have affected the uniformity of data retrieval. The sample size could be quite small, although it is more plentiful than previous studies. Owing to the retrospective type of the study, the patients did not assume the same PPI. However, we demonstrated that this type of medication does not influence the response to tailored therapy, similarly to some previous evidence.^37, 38^ In conclusion, the present report was stimulated by the low number of studies focusing on therapy optimization for refractory NERD according to MII-pH monitoring.^13, 25^ Additionally, some of these investigations did not quantitatively evaluate symptom improvement by a VAS or Likert numeric scale, as well as an agreement on the treatment according to the type of reflux was not homogeneously ascertained. However, our experience suggests that tailored therapy may be the best therapeutic strategy for refractory NERD even if studies on large populations are needed to reach a final consensus for a standardized strategy in clinical practice.

## Materials and methods

### Selection of patients

We retrospectively enrolled NERD patients who had undergone MII-pH monitoring in two Gastroenterology Unit in Southern Italy (University Hospital Policlinico of Bari and 'De Bellis' Hospital of Castellana Grotte) along the period January 2016–March 2017. We selected patients with reflux symptoms, both atypical and typical, who fulfilled criteria for refractoriness, that is, the absence of symptom improvement after a 8-week course of PPI. All patients performed upper endoscopy before MII-pH monitoring and subjects with endoscopic sings of erosive esophagitis were excluded to avoid potential misleading diagnosis of refractory disorder for the presence of inflammation and/or erosion-related complaint.^13^ Similarly, further exclusion criteria were: Barrett esophagus, gastric or duodenal peptic ulcer, esophageal diverticula, history of cancer or surgery of upper gastrointestinal tract, severe heart or lung disorders, diabetes, rheumatologic disorders and psychiatric impairment. We further excluded patients assuming non-steroidal anti-inflammatory drugs, aspirin, oral anticoagulants and other drugs associated with esophageal damage (bisphosphonates, tetracyclines, iron, cancer chemotherapy).

For each patient, the following data were recorded: sex, age, body mass index, alcohol intake, smoking habits and type of typical and atypical symptoms. All patients gave written informed consent and accepted to participate in the study at the moment of personal interview.

### Combined MII-pH monitoring

All patients undergoing MII-pH monitoring had experienced a 2-week washout from PPI. Therefore, the test was performed 'off therapy'.

The test was carried out using the Multichannel Intraluminal Impedance Ambulatory System (Sandhill Scientific, Highlands Ranch, CO, USA) with a 24-h recording. Patients had been in fasting conditions for at least 8 h before the investigation. The recorder was calibrated using buffer solutions at pH 4 and 7. The instrument is provided with a data logger equipped with impedance pH amplifiers as well as a catheter containing 1 pH and 6 impedance channels. The catheter was introduced through the nostril into the esophageal body to record pH at 5 cm and impedance at 3, 5, 7, 9, 15 and 17 cm above the lower esophageal sphincter. Successively, subjects were recommended to continue their usual daily activities and assume meals at normal times. They were suggested to keep upright position during daytime and get a clinostatic position only at bedtime. The data logger also recorded events as symptoms, meals and postural variations.

The test allowed to identify reflux composition as liquid, gas or mixed according to Vela *et al.*^39^ Reflux episodes were characterized by MII-pH monitoring as acid, non-acid and functional according to Zerbib *et al.*^40^

Therefore, NERD patients were subgrouped into three categories according to the following criteria: (i) acid reflux (exposure to pH<4 for at least 1.1% of record time), (ii) non-acid reflux (symptom association probability to pH>4 reflux episodes >95%) and (iii) functional heartburn (no pathologic reflux, with symptom association probability <50%).

### Tailored therapy and definition of response

MII-pH-guided therapy was performed as follows:

1. Patients with acid reflux received PPI at double dose (e.g. omeprazole 20 mg twice daily (b.i.d.) or lansoprazole 30 mg b.i.d. or rabeprazole 20 mg b.i.d. or esomeprazole 40 mg b.i.d. or pantoprazole 40 mg b.i.d.).
2. Patients with non-acid reflux assumed PPI at full dose (e.g. omeprazole 20 mg per day or lansoprazole 30 mg per day or rabeprazole 20 mg per day or esomeprazole 40 mg per day or pantoprazole 40 mg per day) plus alginates one stick four times per day.
3. Patients with functional heartburn were given levosulpiride 75 mg per day divided into three doses.

All above-mentioned treatments were given for 4 weeks before evaluating their effectiveness.

A VAS, largely used in literature^27, 41^ ranging from 0 to 100 mm was administered at least 1 week before the MII-pH monitoring and 4 weeks^13^ after tailored therapy to evaluate overall symptom changing. Responders were defined by VAS improvement of at least 40%, as described by Savarino *et al.*^27^

### Statistical analysis

All continuous variables were tested with the Kolmogorov–Smirnov test, which demonstrated a non-Gaussian distribution. Therefore, nonparametric tests were used. For the comparisons between two paired groups (before/after analysis), the Wilcoxon's matched-pair test was used. When the comparison was carried out among three or more independent groups, the Kruskal–Wallis test and *post hoc* Dunn test for multiple comparisons were performed. Continuous variables were expressed as median and interquartile range. Fisher’s exact test was applied to categorical variables, which were expressed as percentages. If more than three dichotomous variables were compared, a *χ*^2^ test for trend was used. Variables with *P*<0.10 at univariate analysis were analyzed by binomial multivariate regression analysis^42^ aimed to investigate factors predictive of failure of tailored therapy. At multivariate analysis, the estimate of risk was expressed as OR and 95% CI, and values of *P*<0.05 were considered as statistically significant.

## Publisher’s Note

Springer Nature remains neutral with regard to jurisdictional claims in published maps and institutional affiliations.

## Figures and Tables

**Table 1:** Univariate analysis comparing patients responders and non-responders to tailored therapy

	**Non-responders (*N*=11)**	**Responders (*N*=58)**	***P*- value**
Age (mean±s.d.)	47.7±15.9	47.5±15.2	0.97
BMI (mean±s.d.)	23.0±5.0	24.0±3.9	0.54
Delta VAS (mm) (median, interquartile range)	20, 10	50, 20	<0.001
Percentual VAS reduction (%) (median, interquartile range)	22.2, 12.5	66.6, 31.4	<0.001
			
*Proton pump inhibitor*, n *(%)*^a^			0.15
Esomeprazole	2 (40)	11 (23.9)	
Pantoprazole	3 (60)	18 (39.1)	
Omeprazole	0 (0)	10 (21.7)	
Rabeprazole	0 (0)	5 (10.9)	
Lansoprazole	0 (0)	2 (4.4)	
			
Female sex, *n* (%)	9 (91.8)	34 (58.6)	0.19
Smoking habits, *n* (%)	0 (0)	17 (29.3)	0.054
Alcohol consumption, *n* (%)^b^	1 (9.1)	10 (17.2)	0.68
Dysphagia, *n* (%)	2 (18.2)	4 (6.9)	0.24
Heartburn sensation, *n* (%)	7 (63.6)	43 (74.1)	0.48
Epigastric pain, *n* (%)	6 (54.5)	27 (46.6)	0.75
Sensation of 'feeling full quickly', *n* (%)	7 (63.6)	30 (51.7)	0.53
Nausea, *n* (%)	6 (54.5)	14 (24.1)	0.06
Belching, *n* (%)	6 (54.5)	30 (51.7)	1.00
Regurgitation, *n* (%)	6 (54.5)	34 (58.6)	1.00
Vomit, *n* (%)	2 (18.2)	3 (5.2)	0.29
Atypical symptoms, *n* (%)	5 (45.4)	24 (41.4)	1.00
Cough, *n* (%)	1 (9.1)	14 (24.1)	0.43
Globus, *n* (%)	2 (18.2)	3 (5.2)	0.18
Hoarse voice, *n* (%)	3 (27.3)	13 (22.4)	0.71
Ear pain, *n* (%)	0 (0)	1 (1.7)	1.00
Acid reflux, *n* (%)	3 (27.3)	23 (39.6)	0.51
Non-acid reflux, *n* (%)	2 (18.2)	23 (3.96)	0.30
Functional heartburn, *n* (%)	6 (54.5)	12 (20.7)	0.03

Abbreviations: BMI, body mass index; VAS, visual analog scale

a Percentages have been calculated according to the total number of patients assuming a proton pump inhibitor

b Alcohol assumption was defined as >12 g per day for women and >25 g per day for men

**Table 2:** Estimation of risk, expressed as ORs, for failure to tailored therapy, both in univariate and multivariate analysis (binomial logistic regression)

	**Univariate analysis**		**Multivariate analysis**	
	**OR (95% confidence interval)**	***P*-value**	**OR (95% confidence interval)**	***P*-value**
Smoking habits	0.26 (0.07–1.00)	0.054	0.1 (0.002–18.9)	0.99
Nausea	3.77 (0.99–14.27)	0.06	3.67 (0.85–15.88)	0.08
Functional heartburn	4.60 (1.20–17.68)	0.03	3.67 (0.85–15.88)	0.08

Abbreviation: OR, odds ratio

Values with *P*=0.10 at univariate analysis were entered the multivariate analysis, and statistical significance was set at *P*=0.05
